# Specificity of the inhibition of DNA synthesis by extracts from cloned normal, sarcoma-virus-transformed and revertant 3T3 cells.

**DOI:** 10.1038/bjc.1978.280

**Published:** 1978-12

**Authors:** P. Ebbesen, L. Olsson

## Abstract

Extracts containing tissue-specific DNA-inhibitory activity were prepared from normal FL (Swiss) and BALBc3T3 cells, from these cells transformed with sarcoma virus and from revertants cloned from the transformed cell lines. By testing all extracts on all cell lines we found that (1) production of and susceptibility to the inhibitors were decreased in transformed BALB/c cells (2) specificity varied with expression of the transforming genome, as an extract from a given cell line inhibited the growth of its cell of origin, e.g. revertant, more than normal or transformed cells, and (3) there was also a DNA-synthesis stimulator.


					
Br. J. Cancer (1978) 38, 732

SPECIFICITY OF THE INHIBITION OF DNA SYNTHESIS BY EXTRACTS

FROM CLONED NORMAL, SARCOMA-VIRUS-TRANSFORMED

AND REVERTANT 3T3 CELLS

P. EBBESEN AND L. OLSSON

From the Department of Tumor Virus Research, Institute of Medical Ailicrobiology,

University of Copenhagen, DK-2100 Copenhagen 0, Denmiark

Received 17 July 1978  Accepted 28 August 1978

Summary.-Extracts containing    tissue-specific DNA-inhibitory  activity were
prepared from normal FL (Swiss) and BALB/c 3T3 cells, from these cells transformed
with sarcoma virus and from revertants cloned from the transformed cell lines.
By testing all extracts on all cell lines we found that (1) production of and suscepti-
bility to the inhibitors were decreased in transformed BALB/c cells, (2) specificity
varied with expression of the transforming genome, as an extract from a given cell
line inhibited the growth of its cell of origin, e.g. revertant, more than normal or
transformed cells, and (3) there was also a DNA-synthesis stimulator.

REVERSIBLE DNA-inhibitory activity
in extracts from normal and revertant
mouse (FL (Swiss) and BALB/c 3T3)
cells inhibits in vitro DNA synthesis in
the cell of origin. In contrast, extracts
from transformed BALB/c cells, which
are highly malignant in vivo, have no
inhibitory effect on their cell of origin
(Ebbesen et al., 1977). By testing all
extracts on all cell lines we now find that
-both production and susceptibility are
decreased in transformed BALB/c cells,
that the specificity of action of the
extracts varies with expression of the
transforming genome, and that there is
also a compound stimulating DNA syn-
thesis.

MATERIALS AND METHODS

Cells.-Early passages of cloned murine
3T3 FL cells derived from outbred Swiss
mice and 3T3 cells derived from inbred
BALB/c mice, Moloney sarcoma virus-
transformed, sarcoma-genome positive, leu-
kaemia-virus negative (S+L-) 3T3 cells,
Kirsten sarcoma virus-transformed, non-
producer (NP) 3T3 cells, and flat revertants
cloned from the transformed cultures (No-

mura et al., 1972; Fischinger et al., 1974) were
used. Tests for tumour-virus production and
contamination by bacteria and mycoplasm
Ebbesen et al., (1977) were negative.

Preparation   of  extract.-Cells  were
harvested at 50%o confluence. After mechani-
cal disruption the cell fragments were
centrifuged at 105,000 g at 4?C for 1 h, the
supernatant heated to 70?C for 15 min, and
centrifuged at 105,000 g at 4?C for 1 h. The
last supernatant was then separated by
filtration into mol.-wt fractions < 20,000,
20-50,000, 50-100,000, 100-300,000 and
> 300,000. The inhibitory and stimulatory
activity was found in the 20-50,000 fraction,
and this fraction was used throughout
(Ebbesen et al., 1977; Olsson and Ebbesen,
1977). The amount of protein and nucleosides
was determined spectrophototometrically in
each sample, which then was lyophilized
and stored at -70?C. The amount of nucleo-
sides was < 100 w/\rw of the protein in the
extracts used.

IN VITRO test.-The extract from 106
cells was added to a subconfluent culture
with 5 x 105 cells ? 2 x 105 (s.e.) in a
30 cm2 bottle. Following incubation at 37WC
for 18 h, 10 UCi [3H]TdR was added, and 4 h
later the cells were harvested, counted, and

Correspondence: P. Ebbesen, M.D., Department of Tumor Virus Research Institute of Medical Micro-
biology 30, Juliane Maries vej DK-2100 Copenhagen 0, Denmark.

SPECIFICITY OF THE INHIBITION OF DNA SYNTHESIS      733

-*     *   * *

00   I  I  ~   I  I I  I  I

z

-s    Cv b rt _ _b: O  CO *

rI

00     *  *  *  * *

r Ct  00  00 00 C

00Z  I I 0 C tI  0 0s b 4

I

-     *      *  *   *

I-.-       00 0 C 4O _   C9_

C~~I~ ~~000~~0O 000 m

X-  I  I   I

00  C)     m~~~~~~~~~~~~C

_            *               CO
oo     000cq   0  00  --

_   qI  I           I 4 e

00

*    * *  **  *  *            0

00                       m~~~~~

Is  I tI   I       I X I

00       *  * **   *             CO

CD

C)

P 4   Ck  M C 0s tP _           r. 4

X   I   I   I  C)

I0~ C C   - - I

0

00          I co

t      ~*  *     *  * *

00 ccz   00Os  tO  "  00ss : <

I IIII        eI           C)t
00                               t

CO  4

00 tD ? G b X x H M M E X > m t  t 0  > 6

a ?.WH;XtXe~~~~00m mPM 1 1 11

m         p: ~4

CL)
CO
0
0

E

-o -

m

".

00
0

o
CO

C.   .
*__   C

V,a   t
C.o)

C 0  O
~C. C )

?0~

00s ;
oL C)o

C) c
o

0

*y

.)

3

0

CO
CO

00

--

P. EBBESEN AND L. OLSSON

K

o

IQCID

o    I

QJ)

,Q

x

x   O    0

x

O X 0 00

0 0 0 O
000

X xoD xO

x x *   C)
X0 00 0
* x O  x

0  O X   0 00

:r ^

.t      - .

.( -4

C) ce. C4 -.4

?1- 4       P

C)

SL

0o

z

C
C

C

.5

C
C
C

C
C

C ?

C ? ?

If  Ii  II  Ii
ZE?O

734

. IQ

I
t

Q                   I

. 'IQzl-?

CIQ

I                  I

?-q

pq
1-1

I
I
i

I       I

I

i
I

i

?-4          1-
P?

C) ??; -

T -_9

z " E-.q -

I 0 4'? - oc ??Pg 9

E I? -   "         1-0 1-0

, , .14 Cl"., e.11

M . .       "  oo cl,: t-  I

.T E--? =  9  -     -  cq  I

?: V., ^M x -.q ii ?w PL, ,
:c                         I

SPECIFICITY OF THE INHIBITION OF DNA SYNTHESIS

the [3H]TdR incorporation determined. The
reversibility, absence of cytotoxicity and
specificity of the extracts wvere also tested
(Ebbesen et (al., 1977).

RESU LTS

rI'he cross-board study (Tables I and II
shows that the strongest DNA-synthesis
inhibition of a given extract is invariably
found when tested on the cell from which
the extract was obtained. The total
number of cell lines that were signi-
ficantly inhibited in DNA synthesis in
relation to the total number of cell lines
tested was 8/22 (36%) for normal cells,
22/66 (330o) for revertant cells, and 6/33
(1 80) for transformed cells. The total
number of extracts inhibiting DNA syn-
thesis in relation to the total number of
tested extracts from all cell types, was
9/22 (41 %) for extracts from normal cells,
20/66 (300o) for extracts from revertant
cells, and 7/33 (21? o) for extracts from
transformed cells. The tendency not
statistically significant of transformed
cells both to contain low inhibitory
activity and to be less susceptible to
otherwise inhibitory extracts, is specially
pronounced for cell lines derived from
the BALB/c mouse.

Tables I and II further show that some
extracts, when added to certain cell lines,
may stimulate DNA synthesis. The total
number of cell lines stimulated in relation
to the total number of cell lines tested
was 1/22 (4-50o) for normal cells, 10/66
(155%) for revertant cells, and 3/33 (90o)
for transformed cells; and the total
number of stimulatory extracts in relation
to the total number of extracts tested
was 2/22 (90o) for normal cells, 5/66 (8%o)
for revertant cells, and 7/33 (210%) for
transformed cells.

The Figure shows dose-response curves
of typical inhibitory and stimulatory
extracts respectively, tested on 2 cell
lines. Inhibitory extracts had no stimu-
latory effect at any concentration tested.
Stimulatory extract had no inhibitory
effects, as the inhibition observed in

0

0
_n
C

No. of cells used for extract added to each cell culture

FIG. Dose-response curves of the effect on

[3H]TdR incorporation in cells from 2 cell
types treated with extracts from the same
2 cell types. Each point represents the value
of the mean ? s.e.

0-
A*

A-

(9
-0
-A
-A

Extract

from
3Bll
3B 1

SR448
SR448

Target

cells
3Bll

SR448
3Bll

SR448

experiments with stimulatory extracts
was due to increasing cell death as
measured by trypan-dye exclusion. Thus
extract from 5 X 106 cells induced no
increased cell death, whereas extract of
107 and 5 x 107 cells induced 80-90%
cell death. The cytotoxic effect of inhibi-
tory extract was also only observed with
extract doses above 5 x 106 cells and
again with a cytotoxicity level about
80-900o.

DISCUSSION

Transformed K-BALB 23 cells are not
inhibited by their own extract (Ebbesen
et al., 1977) and, as has been shown for
some other malignant cells (Bullough &
Deol, 1975; Rytomaa & Kiviniemi, 1968;
Todaro & De Larco, 1976), these malig-
nant cells are here found "deficient"
both with respect to production of active
inhibitory material and with respect to
sensitivity to the various inhibitorv ex-
tracts tested here.

Each of the 11 different cell extracts

735

736                    P. EBBESEN AND L. OLSSON

studied had its most pronounced inhibi-
tory effect on its cell of origin. Since all
cultures of a given strain were derived
from one another by cloning, this indicates
for the first time that there may be a link
between a certain degree of specificity
and expression of the transforming
genomes. Such a change in specificity
would add a new way of escaping normal
regulation (Lord et al., 1974).

Another unexpected finding was the
stimulatory  effect  exerted  by  some
extract: target-cell combinations. The
inhibitory and stimulatory effects are
most probably due to different compounds,
as different responses were obtained with
different extracts on the same cell type,
and as indicated by the dose-response
curves. Our extracts were harvested
from washed trypsinized cells, but this
does not exclude the possibility that they
contain a mitogen from the calf serum
that was adsorbed to the cells. Calf serum
contains a compound mitogenic for human
fibroblasts, but this mitogen is removed
by ultrafiltration (Houck et al., 1972),
as done by us. Mouse cells, however, are
also stimulated by other serum factors
of unknown molecular weight (Rudland
et al., 1974; Brooks, 1976). The stimula-
tory effect of some, but not all, cell
extracts thus could derive from prefer-
ential binding/uptake of exogenous mito-
gen by some cells, although we favour
the hypothesis that we are dealing with an
enhanced endogenous production of
mitogen.

Our finding of both quantitative and
qualitative (specificity) differences, co-
varying with expression of malignant
phenotype within cultures derived from
each other by cloning, supports the
assumption that such extracts contain
regulatory compounds relevant to cancer

biology, and justifies further work oIn
purification.

This investigation was supported in part by
National Cancer Institute Grant No. 5 ROI Ca 170
39 02, Commission of the Europeain Commuinities
Grant No. 251-77-1 BIO DK, The Daniish Canicer
Society and Dr Rask-Nielsens Fondl.

REFERENCES

BRooKs, R. F. (1976) Regulation of the fibroblast

cell cycle by serum. Nature, 260, 248.

BULLOIUGH, W. S. & DEOL, .1. U. R. (1975) Dermo-

epithelial adhesion andl its effect on epidermal
structure in the mouse. Br. J. Dermtiatol, 92, 417.
EBBESEN, P., OLSSON, L., RUDKOBING, O., HAAHR,

S. & KRISTENSEN, G. (1977) Coirrelation of some
cell functions to transformation/reversion studied
with cloned Moloney and Kirsten sarcoma virus
transformed mouse 3T3 cells. I. IJo vivo malig-
inancy, net outer charge, in vitro migration,
interferon activity, in vitro growth rates and
chalone-like activity. Canicer Res., 37, 4285.

FIscHINGER, P. J., NOMITRA, S., TUTTLE-FuLLER,

X. & DL'NN, K. J. (1974) Revertants of mouse
cells transformed by mturine sarcoma virus. III.
Metastable expression of virus ftunctions in
revertants r etransformecd by muirine sarcoma
virus. 1Virology, 59, 217.

IfOU-CK, J. C., WEIL, R. L. & SARAIA, V. K. (1972)

Evidence for a fibroblast chalonie. Nature, 102,
210.

LORD, B., CERCEK, L., CERCEK, B., SHAH, G.,

DEXTER, T. & LAJTHA, L. (1974) Inhibitors of
haemopoietic cell proliferation. 2: specificity of
action wAithin the haemopoietic syst,em. Br. J.
Cancer, 29, 168.

NOMTTRA, S., FISCHINGER, P. J., MIATTERN, C. F.,

PEEBLES, P. T., BASSIN, R. H. & FRIEDMAN, G. P.
(1972) Revertants of mouse cells transformed by
murine sarcoma viruis. I. Characterization of flat
and transformed sublines without, a Irescuable
murine sarcoma viIrus. Virology, 50, 51.

OLSSON, L. & EBBESEN, P. (1977) Ageing decreases

the activity of epidermal G1 and G2 inhibitors in
mouse skin, independent of grafting on old or
young recipients. Exp. Gerontol., 12, 59.

RUlDLAND, P. S., SEIFERT, W. & GoSPODAROWICZ, D.

(1974) Growth control in culturedl mouse fibro-
blasts: induction of pleiotypic and mitogenic
r esponses by a purified growth factor. Proc.
Natl Acad. Sci., U.S.A., 71, 2600.

RYTOMAA, T. & KIVINIEMI, K. (1968) Control of

RNA duplication in rat clloroleukemia by means
of the granulocytic chalone. Eur. J. Catncer, 4,
595.

TODARO, G. J. & DE LARCO, J. E. (1976) Trans-

formation by murine and feline saicoma viruses
specifically blocks binding of epicleimal gi-owth to
cells. Nature, 264, 26.

				


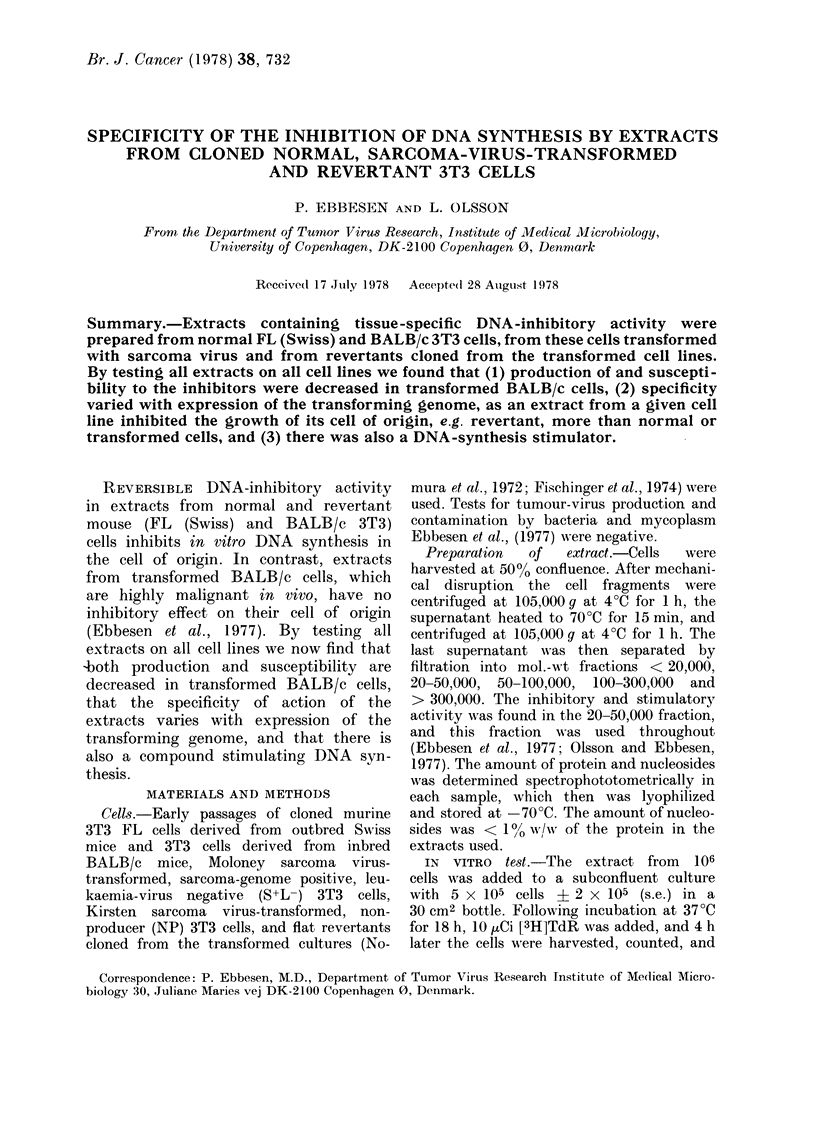

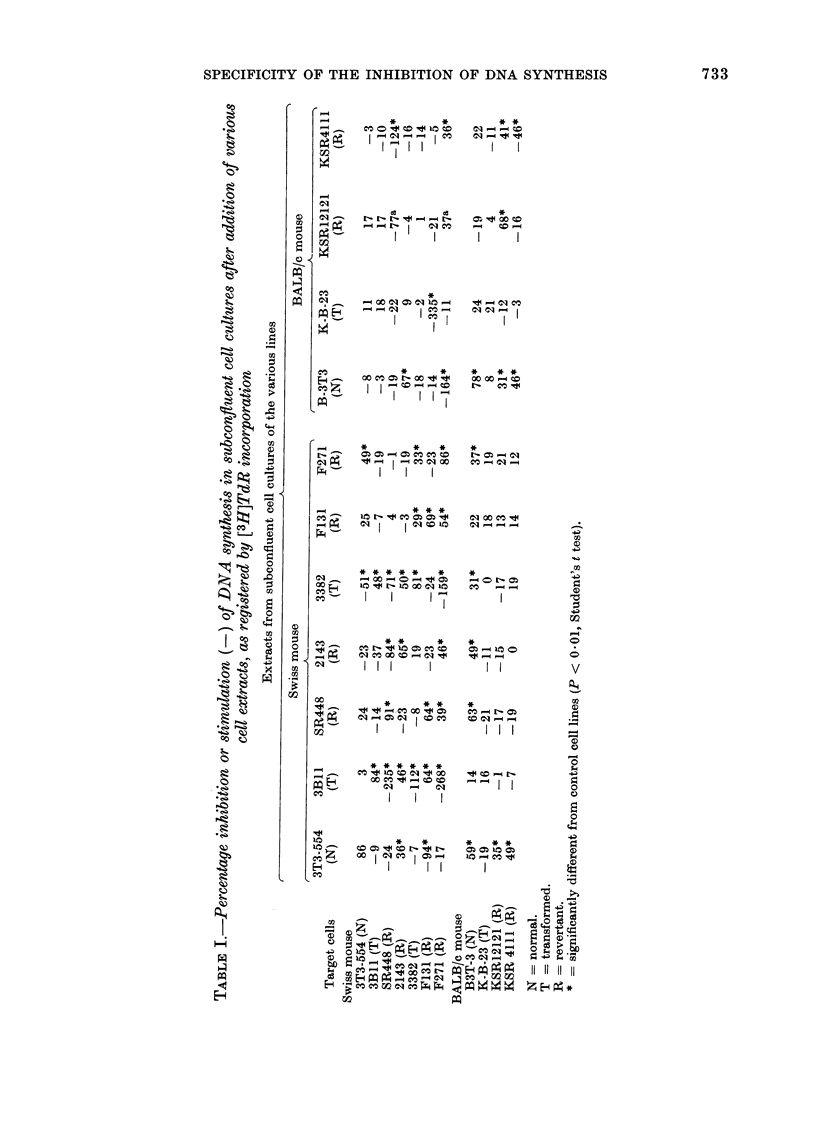

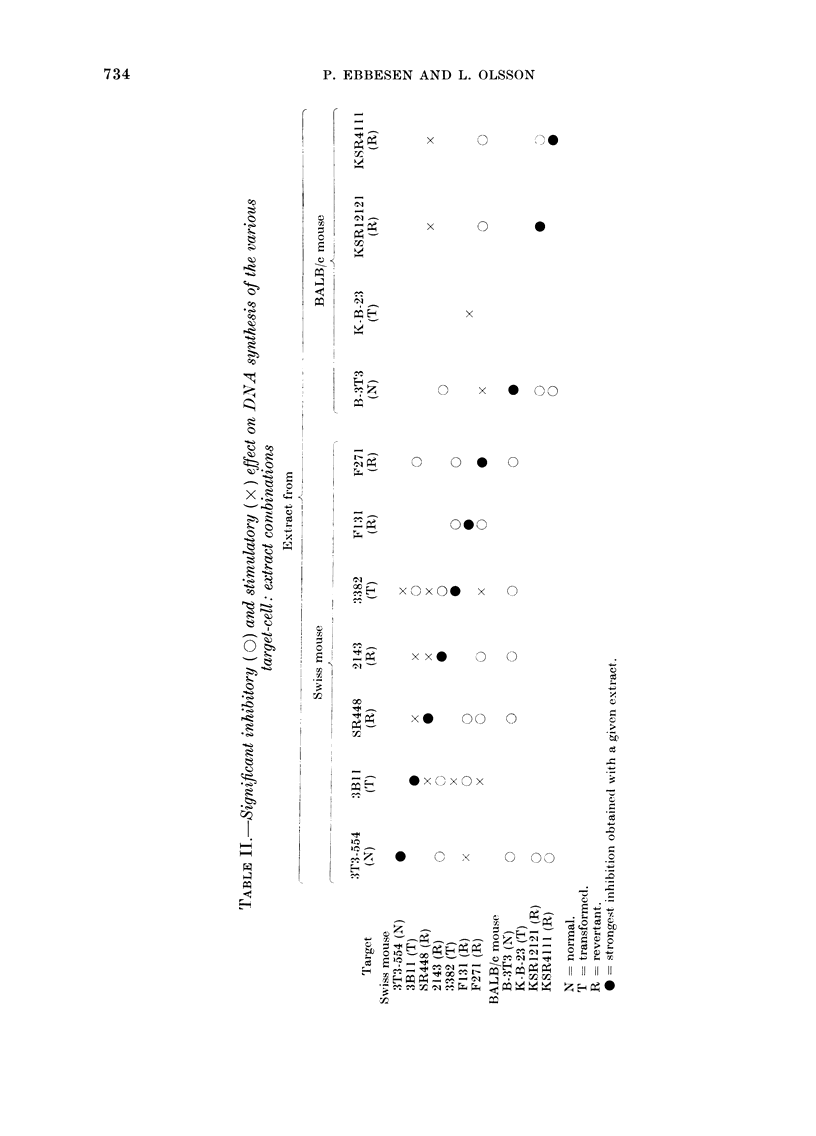

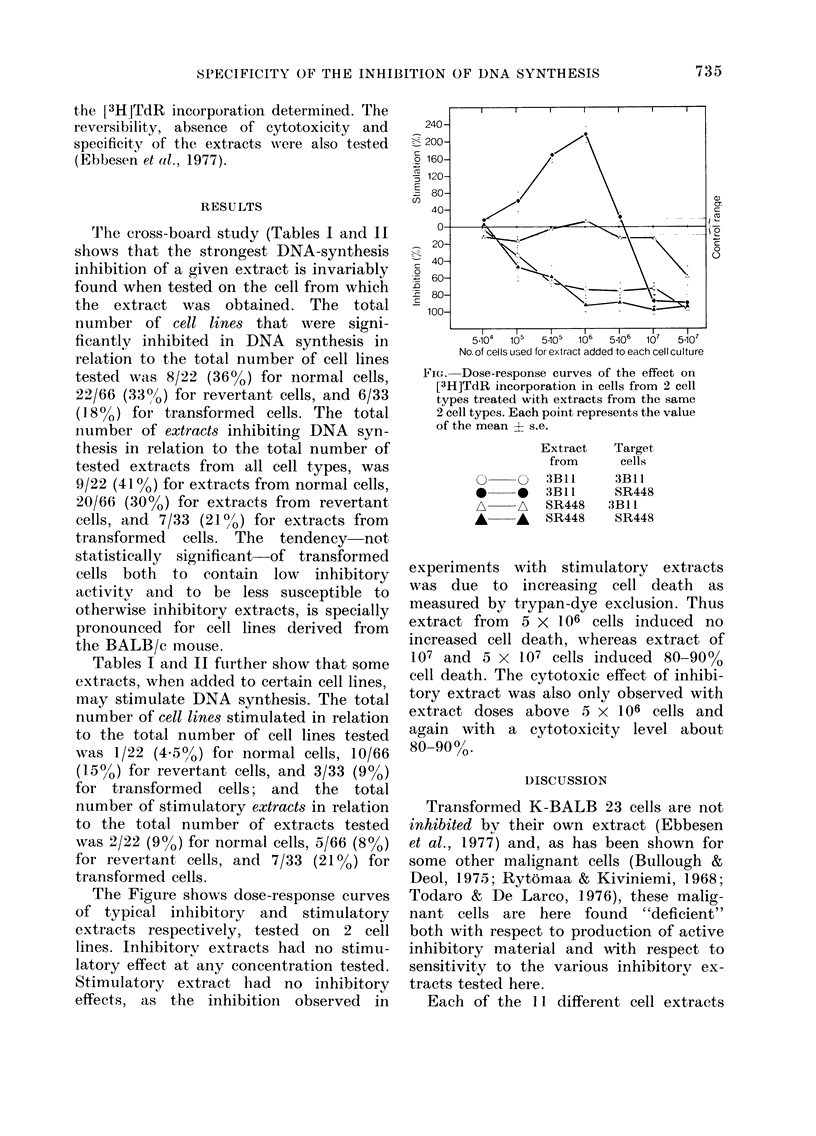

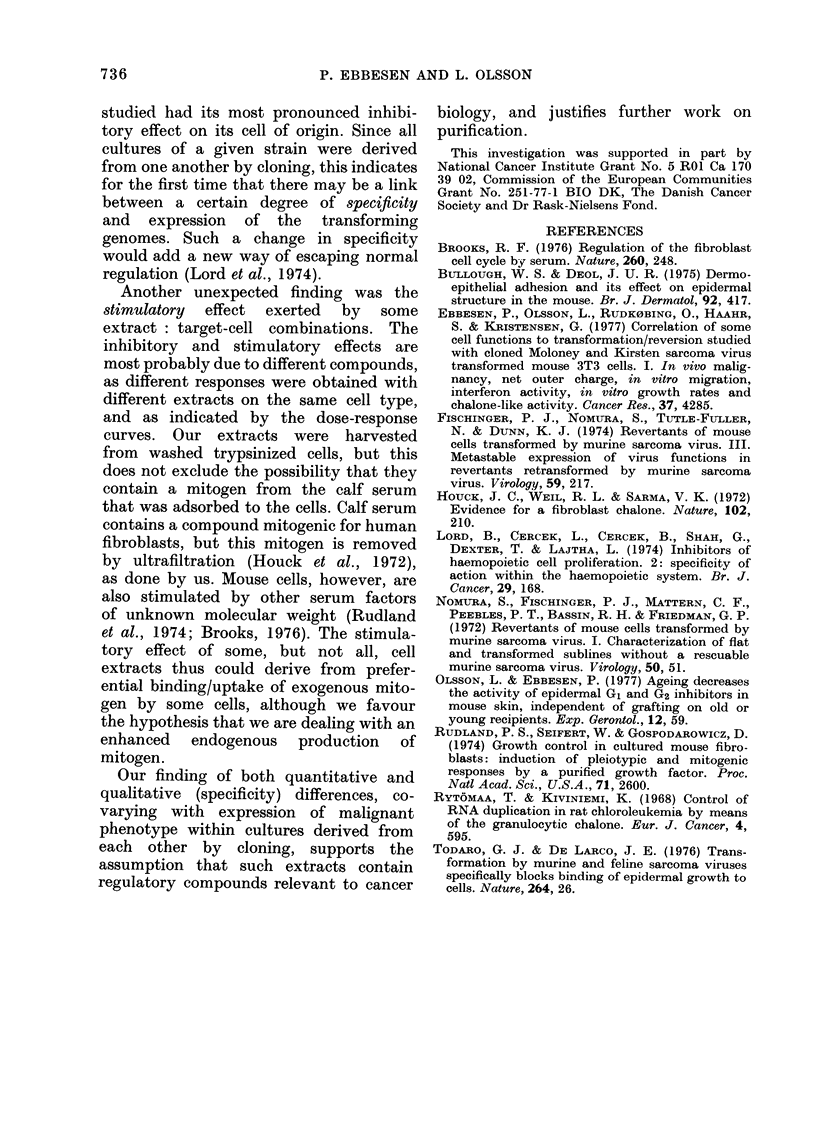

